# Spin-orbit interaction induced anisotropic property in interacting quantum wires

**DOI:** 10.1186/1556-276X-6-213

**Published:** 2011-03-11

**Authors:** Fang Cheng, Guanghui Zhou, Kai Chang

**Affiliations:** 1 Department of Physics and Electronic Science, Changsha University of Science and Technology, Changsha 410076, China; 2 SKLSM, Institute of Semiconductors, Chinese Academy of Sciences, P. O. Box 912, Beijing 100083, China; 3 Key Laboratory (Educational Ministry) for Low-Dimensional Structures and Quantum Manipulation, Hunan Normal University, Changsha 410081, China

## Abstract

We investigate theoretically the ground state and transport property of electrons in interacting quantum wires (QWs) oriented along different crystallographic directions in (001) and (110) planes in the presence of the Rashba spin-orbit interaction (RSOI) and Dresselhaus SOI (DSOI). The electron ground state can cross over different phases, e.g., spin density wave, charge density wave, singlet superconductivity, and metamagnetism, by changing the strengths of the SOIs and the crystallographic orientation of the QW. The interplay between the SOIs and Coulomb interaction leads to the anisotropic dc transport property of QW which provides us a possible way to detect the strengths of the RSOI and DSOI.

PACS numbers: 73.63.Nm, 71.10.Pm, 73.23.-b, 71.70.Ej

## Introduction

All-electrical manipulation of spin degree of freedom is one of the central issues and the ultimate goal of spintronics field. The spin-orbit interaction (SOI) is a manifestation of special relativity. An electric field in the laboratory frame can transform into an effective magnetic field in the moving frame of electron and consequently leads to electron spin splitting. Therefore, the SOI provides us an efficient way to control electron spin electrically and has attracted tremendous interest because of its potential application in all-electrical spintronic devices [1,2]. The spin degeneracy can be lifted by applying magnetic field to break the time-reversal symmetry and/or by applying electric fields to break the spatial inversion symmetry. However, the latter could be more easily realized in spintronics devices. In semiconductors the spatial inversion symmetry can be broken by the structural inversion asymmetry and bulk crystal inversion asymmetry, named, Rashba SOI (RSOI) and Dresselhaus SOI (DSOI), respectively [3,4]. Usually, the RSOI in semiconductor quantum well is much stronger than that of DSOI, and therefore, most of the previous theoretical and experimental studies focused on the RSOI and its consequence on the spin transport properties in two-dimensional electron gas (2DEG) [5-7]. In thin quantum wells, the strength of the DSOI is comparable to that of the RSOI since the strength of the DSOI depends significantly on the thickness of quantum wells. The interplay between the RSOI and DSOI leads to interesting phenomena in 2DEG, e.g., the anisotropic photogalvanic effect [8], and the persistent spin helix [9]. However, the interplay between the RSOI and DSOI in quantum wires (QWs) remains relatively unexplored.

Very recently, the anisotropic behavior of transport property in semiconductor QWs was proposed to detect the relative strength between the RSOI and DSOI in a quasi-one-dimensional (Q1D) semiconductor QW system [10,11]. However, the effect of the Coulomb interaction on the transport property is not addressed. Since the Coulomb interaction becomes very strong in Q1 D electron systems where electrons are strongly correlated, and therefore the conventional Fermi liquid theory breaks down. There are no fermionic quasi-particle in Q1 D electron gas, and the elementary excitations are bosonic collective charge and spin fluctuations with different propagating velocities. The Luttinger liquid (LL) theory [12] is of fundamental importance because it is one of a very few strongly correlated non-Fermi liquid systems that can be solved analytically. The LL displays very unique properties, e.g., the spin and charge separation and the power-law behavior of the correlation functions. The unique behavior was observed experimentally in many Q1 D systems, for instance, narrow QW formed in semiconductor heterostructures [13], carbon nanotube [14], graphene nanoribbon [15], as well as the edge states of the fractional Quantum Hall liquid [16]. Recent studies have found that the RSOI would lead to the mixing between the spin and charge excitations in QW with the RSOI alone [17-20]. It is interesting to study the interplay between the RSOI and DSOI on the ground state and transport property of QWs in the presence of the Coulomb interaction.

In this study, based on the LL theory, we study the effect of the interplay between the Coulomb interaction and SOIs on the electron ground state and transport property of an interacting QW oriented along different crystallographic directions in different planes. The electron ground state can display the transitions among the different phases, e.g., the spin density wave (SDW), charge density wave (CDW), singlet superconductivity (SS), and metamagnetism (MM), by tuning the crystallographic plane and orientation of the QW, the strengths of SOIs and the Coulomb interaction. The anisotropy of the dc conductivity of interacting QW is induced by the interplay between the Coulomb interaction and SOIs, which could be used for detecting the strengths of the RSOI and DSOI.

## Theory

**Figure 1:**
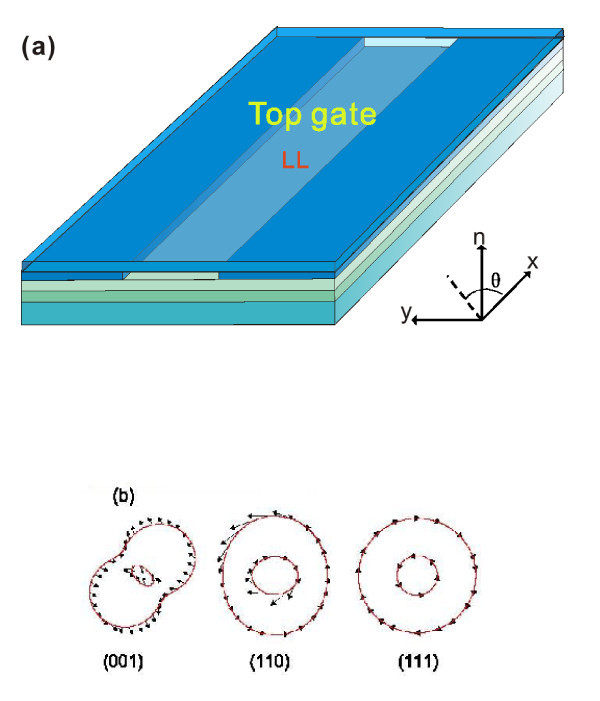
**Schematic diagram of semiconductor QW, the constant energy surfaces and the spin orientations of 2DEG**. **(a) **Schematic diagram of semiconductor QW which oriented along the crystallographic direction *θ *with respect to [100] axis in the crystallographic planes (001), (110), and (111). *n *is the unit vector along the normal direction of the crystallographic plane. **(b) **The constant energy surfaces and the spin orientations of 2DEG in (001), (110), and (111) planes.

The Q1 D QW system is shown schematically in Figure 1a where electrons are confined laterally and move freely along the *x*-axis. The RSOI can be generated in the region below the top gate, and the DSOI always exists in the conventional zincblende semiconductors lacking the spatial inversion symmetry, e.g., GaAs, InAs, and InSb. The Hamiltonian of noninteracting electrons in a Q1 D QW oriented along different crystallographic directions in (001) plane is [11](1)

where *θ *is the angle between the orientation of the QW and the [100] axis, m* is the electron effective mass, *σ_i _*(*i *= *x, y, z*) are the Pauli matrices, and *α *and *β *are the strengths of the RSOI and DSOI, respectively. The linearized non-interacting electron Hamiltonian of the QW with both RSOI and DSOI is [17,20](2)

where the operators *ψ_γs_*(*γ *= -1(*L*),1(*R*); *s *= -1(↓),1(↑)) annihilate spin-down (↓) or spin-up (↑) electrons near the left (L) and right (*R*) Fermi points, respectively.  are the four different Fermi velocities, where *v*_F _is the bare Fermi velocity of right- and left-moving non-interacting electrons, , usually *δv *≪ *v*_F_. Note that the RSOI and DSOI split the spin-up and spin-down subbands and make the electron Fermi velocities become orientation dependent. The total Hamiltonian of the system including the Coulomb interaction is *H *= *H*_0 _+ *H*_int_, where(3)

The Umklapp scattering process is neglected because the Fermi energy in QWs formed in semiconductor heterostructure is far from the half-filled case, and the electron-electron backscattering can be negligible for a sufficiently long interacting region [17]. Using the bosonization technique [21], the Hamiltonian becomes(4)

Where *ϑ_ρ _*and *ϑ_σ _*are the phase fields for the charge and spin degrees of freedom, respectively, and Π*_ρ _*and Π_σ _are the corresponding conjugate momenta. *v_ρ _*and *v_σ _*are the propagation velocities of the decoupled charge and spin-collective modes in the absence of the SOIs (*δv *= 0), respectively.

For a QW embedded in (110) plane, the Hamiltonian of the noninteracting electrons reads [11](5)

where the parameters *α, β*, and *θ *are the same as in Equation (1). The dominant differences are (1) the Fermi velocity is different , where ; (2) the effective magnetic field induced by the DSOI is perpendicular to (110) plane, which could generate an out-of-plane spin-polarized current; and (3) the anisotropy is mainly determined by the DSOI. These differences would lead to the distinct anisotropic behavior of QWs embedded in (110) plane. For (111) plane, the DSOI shows the same formulism as the RSOI; the Hamiltonian is  which does not contain any *θ*-dependent term, and therefore the anisotropic behavior disappear. This isotropic character can be easily seen from the constant energy surface (see Figure 1b). We will not discuss the (111) plane any more.

The correlation functions at zero temperature in an interacting QW in the presence of the SOIs behave as , and the exponents *α_i_*'s are determined by [18](6)

Here(7)

Where , and(8)

are the propagation velocities of coupled collective modes that depend on the crystallographic orientation *θ*. From the above equations, one can see that the interplay between the RSOI and DSOI will lead to anisotropy, i.e., the dependence on the crystallographic direction. This is the dominant difference between this work and the previous study [18]. Interestingly, for a QW in (001) plane, the spin and charge excitations can be decoupled again when *θ *= 3*π*/4 and *α *= *β*, *θ *= *π*/4 and *α *= -*β*, since the coupling between the spin and charge excitation disappear when *δv *= 0 (see the third term in Equation (4)).

Considering a point-like density-density interaction for the electron-electron interaction [22,23], we have *v*_*ρ*,*σ *_= *v*_F_/*K_ρ,σ _*and the parameter *K*_*ρ*/*σ *_being defined as , where *g *= 2*V*(*q *= 0)/*ħπv*_F _with *V *(*q *= 0) is the electron-electron interaction potential. We consider an infinitely long interacting QW, i.e., a homogenous interacting QW, driven by a time-dependent electric field *E*(*x*, *t*), which could be realized by applying a microwave radiation.  describes the effect of the ac electric field on the charge excitation. The total Hamiltonian becomes *H *= *H*_0 _+ *H*_int _+ *H*_ac_. By minimizing the action functional of such a Q1 D system, we get the equation of motion and consequently obtain the non-local charge conductivity using the linear response theory:(9)

## Numerical results and discussions

**Figure 2:**
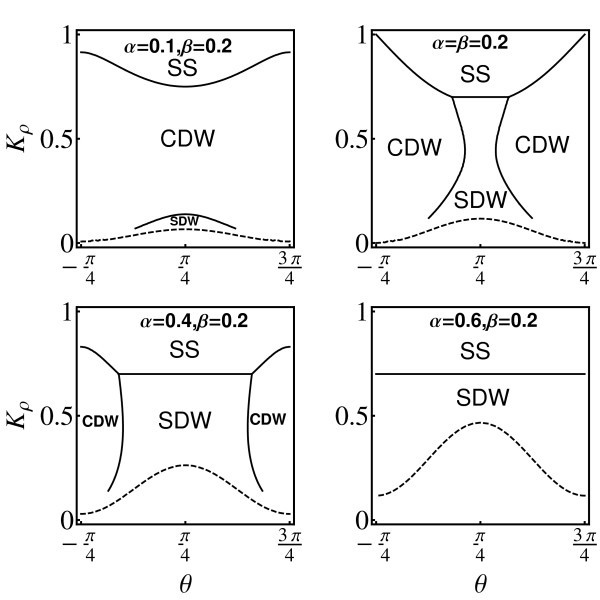
**Phase diagram of the ground state of Q1 D electron gas in a QW embedded in (001) plane for *v_ρ _*= 1.2*v*_F_, *v_σ _*= 0.8*v*_F_, *K_σ _*= 0.7 for different values of the strengths of the RSOI *α *and DSOI *β *(in units of *ħv*_F_)**. *θ *is the crystallographic direction. The regions below the dotted lines (*δv *> *δv_σ_*) indicate the MM phase.

First, we study how the interplay among the Coulomb interaction, the RSOI, and the DSOI affects the ground state of Q1 D electron gas. It is worth to note that if one does not assume any specific form for electron-electron interactions, then all four parameters *v_ρ_*(*σ*) and *K_ρ_*(*σ*) (or equivalently *g*_2||_, *g*_2⊥_, *g*_4|| _and *g*_4⊥_) are independent [18]. Figure 2 describes the phase diagram of Q1 D QW embedded in (001) plane as function of the strength of the Coulomb interaction *K_p _*and crystallographic orientations *θ *for the different strengths of SOIs *α *and *β *when *K_σ _*= 0.7. We should stress that the different phases are determined by which correlation function decays most slowly for |*x*| → ∞ when the other correlation functions become negligible. We define the phase by the dominant correlation function, e.g., CDW or SDW. Here the CDW and SDW are actually not the pure charge and spin fluctuations, but a mixed state of them induced by the SOIs. Interestingly, one can see that the ground state of the system displays a crossover between the CDW and SDW by tuning the crystallographic orientation for the fixed parameters, e.g., *K_ρ_*, *α*, and *β*. Note that the ground state of Q1 D electron system in the presence of RSOI alone cannot be affected by tuning the crystallographic orientation [18]. We find that there are crossovers of the ground state of Q1 D electron gas between CDW and SDW when we tune the strengths of the Coulomb interaction and the SOIs. For a fixed strength of the DSOI, the CDW phase region will be squeezed and die away gradually, with increase in the strengths of the RSOI. The ground state of the system is always SDW at strong RSOI for the arbitrary orientation of the QW. On the contrary, as the strength of the RSOI decreases for a given strength of the DSOI, the CDW phase region expands, and the ground state of the system becomes the CDW eventually. When *δv *>*δv_σ_*, i.e., in the region below the dotted line, the ground state is the so-called MM phase, which was earlier observed in the Q1 D systems, e.g., Ba_3_Cu_2_O_4_Cl_2 _[24], and attributed to the next-nearest-neighbor coupling in the XXZ model [25]. By tuning the parameters properly, the ground state of the system exhibits the crossovers among the SDW, CDW, SS, and MM.

**Figure 3:**
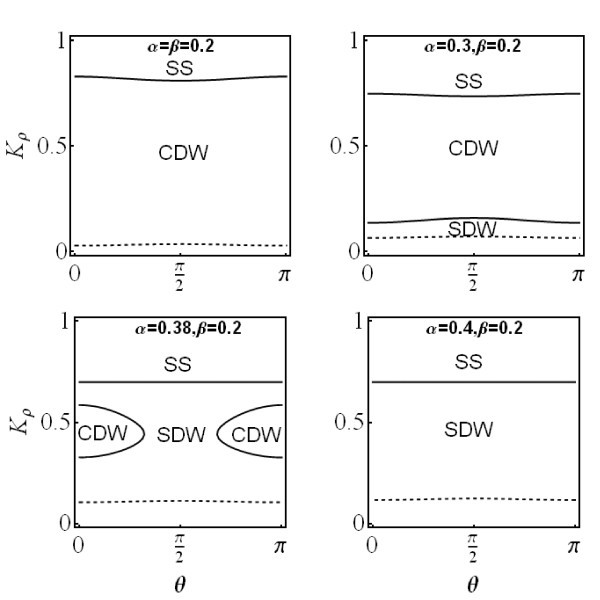
**Phase diagram of the ground state of Q1 D electron gas in a QW embedded in (110) plane**. The same as Figure 2, but for a QW embedded in (110) plane.

The phase diagram of the ground state for the QW embedded in (110) plane is very different from that of the QW in (001) plane for the same parameters *v_ρ_*, *v_σ _*and *K_σ_*. Tuning the strength of the RSOI at a fixed strength of the DSOI, the ground state can also transit from CDW to SDW, but in very narrow region in the *K_ρ _*- *θ *space (see Figure 3). This is because the anisotropy is weaker compared to that of the QW in (001) plane.

**Figure 4:**
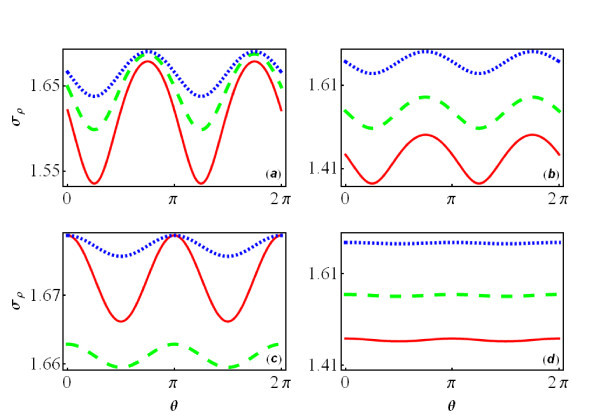
**The dc conductivity dependence of the angle *θ***. The dc conductivity (in units of *e*^2^*/h*) of electron in an infinitely long-interacting QW as a function of the angle *θ *with a fixed strength of Coulomb interaction *g *= 0.4 **(a) **for (001) plane, **(c) **for (110) plane, where the solid line (red online) is for *α *= 0.2 and *β *= 0.4; the dashed line (green online) for *α *= 0.3 and *β *= 0.2; and the dotted line (blue online) for *α *= 0.2 and *β *= 0.2; **(b, d) **is the same as **(a, c)**, but with fixed SOI strengths *α *= 0.2 and *β *= 0.2 for different strengths of Coulomb interaction. **(b) **is for (001) plane and **(d) **for (110) plane, where the dotted line (blue online) corresponds to *g *= 0.4; the dashed line (green online) to *g *= 0.6; and the solid line (red online) to *g *= 0.

Next, we turn to study the transport property of electron in an interacting QW. In the dc case, Equation (9) shows that the charge conductivity of an infinitely long interacting QW for the different parameters *g *and *δv*. For the noninteracting QW structure, there is only single transverse mode, and thus no anisotropy of the dc conductivity can be found. This result can also be obtained from Equation (9) by taking *g *= 0, i.e., non-interacting case, ; we easily obtain *σ_ρ _*= 2*e*^2^*/h*. When the Coulomb interaction is included but without the SOIs, i.e., *α *= *β *= 0, we can obtain an isotropic conductivity *σ_ρ _*= 2*K_ρ_e*^2^*/h *from Equation (9). The spin and charge excitations propagate independently at the velocities *v_ρ _*and *v_σ _*in this case. In the presence of both the Coulomb interaction and the SOIs, the SOIs mix the spin and charge excitations, leading to anisotropic velocities of the collective excitations *u*_1 _and *u*_2 _(see Equation 8), or equivalently the anisotropic interaction parameter , consequently resulting in the anisotropic conductivity . Therefore, the dc conductivity of the infinitely long-interacting QW depends sensitively on the crystallographic direction *θ *of the QW, i.e., the anisotropic transport behavior (see Figure 4). Interestingly, the dc conductivity oscillates with varying the angle *θ *with a periodicity *π*. With increasing the strengths of the DSOI and/or RSOI (see Figure 4a) or the strengths of the Coulomb interaction (see Figure 4b), the oscillation of the conductivity for the infinitely long-interacting QW becomes stronger. This is because the anisotropy of the interaction parameter  depends on the anisotropic velocities of the mixed spin and charge excitations, which increases when the strengths of the SOIs or the Coulomb interaction increase.

For an interacting QWs embedded in (110) plane, the anisotropy of the dc conductivity becomes different (see Figure 4c,d): (1) The RSOI becomes less important for the anisotropy of the dc conductivity comparing with that in (001) plane, the increase of the strength of RSOI only affects the anisotropy slightly (see the dashed green line and dotted blue line in Figure 4c), since the anisotropy is mainly determined by the DSOI. This can be understood from the formulism of the Fermi velocities , where . It is noted that for (001) plane, the anisotropy of dc conductivity will disappear with the RSOI or DSOI alone, while for (110) plane, the anisotropy will disappear only when the DSOI is absent, which can be understood from the expression of *δv*. (2) The effect of the Coulomb interaction on the anisotropy of dc conductivity is weakened significantly when compared to that in (001) plane (see Figure 4b,d).

Finally, we discuss how to detect the relative strength of the SOIs utilizing the anisotropic dc conductivity. We consider a QW embedded in (001) plane. One can tune the strength of RSOI by adjusting the gate voltage. For a QW oriented along the  axis, i.e., *θ *= 3*π*/4 When *α *= *β *the dc conductivity becomes *σ_ρ _*= 2*K_ρ_e*^2^/*h*, where the Coulomb interaction parameter *K_ρ _*can be deduced from the experiments which ranges from 0.4 to 0.7 in semiconductor QWs [26,27]. Therefore, one determines the relative strength of the RSOI and DSOI from the dc conductivity. In our calculation, we take the electron effective mass *m *= 0.067*m_e_*, the strengths of SOIs *α *and *β *are about 1 × 10^-11 ^eVm which is the typical strength of SOI in conventional semiconductors [28]. Electrons only occupy the lowest subband in GaAs QW assuming the Fermi wavevector is *k*_F _= 0.01 nm^-1^.

## Conclusions

In conclusion, we investigate theoretically the effect of the interplay between the Coulomb interaction and the SOIs on the electron ground state and charge transport property of interacting QWs oriented along different crystallographic directions in different planes. We find that the ground state of electrons in the QWs can transit among the different phases, e.g., the SDW, CDW, SS, and MM, by tuning the plane and orientation of the QW, the strengths of SOIs and the Coulomb interaction. The anisotropy of the dc conductivity in an interacting QW is induced by the interplay between the Coulomb interaction and SOIs. This anisotropy enables us to detect the strengths of RSOI and DSOI, which are very important for the comprehensive understanding of spin decoherence and constructing all-electrical spintronic device.

## Abbreviations

DSOI: Dresselhaus spin-orbit interaction; QWs: quantum wires; RSOI: Rashba spin-orbit interaction.

## Competing interests

The authors declare that they have no competing interests.

## Authors' contributions

FC formulated the theory and carried out the calculation. GHZ participated in the interpretation of the results. KC conceived the idea, formulated the theory and participated in the interpretation of the results. All authors read and approved the final manuscript.

This study was supported by the NSFC 10947134, 10974052; the National Basic Research Program of China (2010CB933700); the KLLDQSQC QSQC0901 (Hunan Normal University), Scientific Research Fund of Hunan Provincial Education Department 09C061; and the construct program of the key discipline in Changsha University of Science and Technology.

## References

[B1] žutić I Fabian J Sarma SD Spintronics: Fundamentals and applications Rev Mod Phys 2004 76 323

[B2] Winkler R Spin-Orbit Coupling Effects in Two-Dimensional Electron and Hole Systems. Springer Tracts in Modern Physics 2003 Berlin: Springer and the references therein

[B3] Rashba EI Properties of semiconductors with an extermum loop .1. syclotron and combinational resonance in a magnetic field perpendicular to the plane of the loop Sov Phys Solid State 1960 2 1109 Rashba EI, Sherman EYa: Spin-orbital band splitting in symmetric quantum wells, *Phys Lett A *1988,**129**:175

[B4] Dresselhaus G Spin-Orbit Coupling Effects in Zinc Blende Structures Phys Rev 1955 100 580 10.1103/PhysRev.100.580

[B5] Luo J Munekata H Fang FF Stiles PJ Effects of inversion asymmetry on electron energy band structures in GaSb/InAs/GaSb quantum wells Phys Rev B 1990 41 7685 10.1103/PhysRevB.41.7685 9993064

[B6] Hu CM Nitta J Akazaki T Takayanagai H Osaka J Pfeffer P Zawadzki W Zero-field spin splitting in an inverted *In*_0.53_*Ga*_0.47_*As/In*_0.52_*Al*_0.48_*As *heterostructure: Band nonparabolicity influence and the subband dependence Phys Rev B 1999 60 7736 10.1103/PhysRevB.60.7736

[B7] Koga T Nitta J Akazaki T Takayanagi H Rashba Spin-Orbit Coupling Probed by the Weak Antilocalization Analysis in InAlAs/InGaAs/InAlAs Quantum Wells as a Function of Quantum Well Asymmetry Phys Rev Lett 2002 89 046801 10.1103/PhysRevLett.89.046801 12144493

[B8] Ganichev SD Bel'kov VV Golub LE Ivchenko EL Schneider P Giglberger S Eroms J De Boeck J Borghs G Wegscheider W Weiss D Prettl W Experimental Separation of Rashba and Dresselhaus Spin Splittings in Semiconductor Quantum Wells Phys Rev Lett 2004 92 256601 10.1103/PhysRevLett.92.256601 15245041

[B9] Koralek JD Weber CP Orenstein J Bernevig BA Zhang SC Mack S Awschalom DD Emergence of the persistent spin helix in semiconductor quantum wells Nature 2009 458 610 10.1038/nature07871 19340077

[B10] Scheid M Kohda M Kunihashi Y Richter K Nitta J All-Electrical Detection of the Relative Strength of Rashba and Dresselhaus Spin-Orbit Interaction in Quantum Wires Phys Rev Lett 2008 101 266401 10.1103/PhysRevLett.101.266401 19113779

[B11] Wang M Chang K Wang LG Dai N Peeters FM Crystallographic plane tuning of charge and spin transport in semiconductor quantum wires Nanotechnology 2009 20 365202 10.1088/0957-4484/20/36/365202 19687557

[B12] Luttinger JM An Exactly Soluble Model of a Many-Fermion System J Math Phys 1963 4 1154 10.1063/1.1704046

[B13] Tarucha S Honda T Saku T Reduction of quantized conductance at low temperatures observed in 2 to 10 m-long quantum wires Solid State Commun 1995 94 413 Levy E, Tsukernik A, Karpovski M, Palevski A, Dwir B, Pelucchi E, Rudra A, Kapon E, Oreg Y: Luttinger-Liquid Behavior in Weakly Disordered Quantum Wires, *Phys Rev Lett *2006, **97**:196802 10.1016/0038-1098(95)00102-6

[B14] Yacoby A Stormer HL Wingreen NS Pfeiffer LN Baldwin KW West KW Nonuniversal Conductance Quantization in Quantum Wires Phys Rev Lett 1996 77 4612 10.1103/PhysRevLett.77.4612 10062582

[B15] Zarea M Sandler N Nonuniversal Conductance Quantization in Quantum Wires Phys Rev Lett 2007 99 256804 10.1103/PhysRevLett.99.256804 18233545

[B16] Chang AM Pfeiffer LN West KW Observation of Chiral Luttinger Behavior in Electron Tunneling into Fractional Quantum Hall Edges Phys Rev Lett 1996 77 2538 10.1103/PhysRevLett.77.2538 10061979

[B17] Moroz AV Samokhin KV Barnes CHW Theory of quasi-one-dimensional electron liquids with spin-orbit coupling Phys Rev B 2000 62 16900 10.1103/PhysRevB.62.16900 10990636

[B18] Iucci A Correlation functions for one-dimensional interacting fermions with spin-orbit coupling Phys Rev B 2003 68 075107 10.1103/PhysRevB.68.075107

[B19] Gritsev V Japaridze GI Pletyukhov M Baeriswyl D Competing Effects of Interactions and Spin-Orbit Coupling in a Quantum Wire Phys Rev Lett 2005 94 137207 10.1103/PhysRevLett.94.137207 15904028

[B20] Yu Y Wen YC Li JB Su ZB Chui ST Luttinger liquid with strong spin-orbital coupling and Zeeman splitting in quantum wires Phys Rev B 2004 69 153307 10.1103/PhysRevB.69.153307

[B21] Gogolin AO Nersesyan AA Tsvelik AM Bosonization and Strongly Correlated Systems 1998 Cambridge: Cambridge University Pres

[B22] Dolcini F Trauzettel B Safi I Grabert H Transport properties of single-channel quantum wires with an impurity: Influence of finite length and temperature on average current and noise Phys Rev B 2005 71 165309 10.1103/PhysRevB.71.165309

[B23] Cheng F Zhou GH Transport properties for a Luttinger liquid wire in the presence of a time-dependent impurity Phys Rev B 2006 73 125335 10.1103/PhysRevB.73.125335

[B24] Eckert D Ruck K Wolf M Krabbes G Müller K-H Magnetic behavior of the low-dimensional compounds *Ba*_2_*Cu*_3_*O*_4_*Cl*_2 _and *Ba*_2_*Cu*_2_*O*_4_*Cl*_2_ J Appl Phys 1998 83 7240 10.1063/1.367613

[B25] Gerhardt C Mütter K-H Kröger H Metamagnetism in the XXZ model with next-to-nearest-neighbor coupling Phys Rev B 1998 57 11504 10.1103/PhysRevB.57.11504

[B26] Levy E Tsukernik A Karpovski M Palevski A Dwir B Pelucchi E Rudra A Kapon E Oreg Y Luttinger-Liquid Behavior in Weakly Disordered Quantum Wires Phys Rev Lett 2006 97 196802 10.1103/PhysRevLett.97.196802 17155649

[B27] Steinberg H Barak G Yacoby A Pfeiffer LN West KW Halperin BI Le Hur K Charge fractionalization in quantum wires Nat Phys 2008 4 116 and references therein 10.1038/nphys810

[B28] Yang W Chang K Nonlinear Rashba model and spin relaxation in quantum wells Phys Rev B 2006 74 193314 10.1103/PhysRevB.74.193314

